# Improving soybean seed oil without poor agronomics

**DOI:** 10.1093/jxb/eraa407

**Published:** 2020-12-30

**Authors:** Miguel Alfonso

**Affiliations:** Department of Plant Nutrition, EEAD-CSIC, Avda de Montañana, Zaragoza, Spain

**Keywords:** Crop yield seed oil, soybean, stearic acid, stearoyl-ACP desaturase (SACPD), TILLING

## Abstract

This article comments on:

**Lakhssassi N, Zhou Z, Liu S, Piya S, Cullen MA, El Baze A, Knizia D, Patil GB, Badad O, Embaby MG, Meksem J, Lakhssassi A, Ghazaleh A, Hewezi T, Meksem K**. 2020. Soybean TILLING-by-sequencing^+^ reveals the role of novel *GmSACPD* members in unsaturated fatty acid biosynthesis while maintaining healthy nodules. Journal of Experimental Botany **71**, 6969–6987.

**Manipulating seed oil content and composition is a major goal for plant biotechnologists. However, some of these modifications generate undesired effects, affecting plant growth and yield, and, thereby, decreasing market value. In this work,** Lakhssassi *et al.* (2020) **describe the application of an improved TILLING technique (TILLING-by-sequencing** ^**+**^ **) that was successful in obtaining soybean lines with mutations in novel members of the *GmSACPD* family. These lines showed higher stearic acid levels without compromising nodulation and growth yield. This work shows how powerful the joint utilization of conventional TILLING techniques and next-generation sequencing can be, helping breeders and biotechnologists to overcome metabolic bottlenecks or undesirable growth phenotypes.**

## Engineering plant seed oils is a major goal of plant biotechnology

Plant seed oils are major renewable resources from nature that can be easily extracted in a cost-effective manner. These oils can be used in multiple applications including their edible use (for cooking or margarines) but also as basic components of lubricants, surfactants, paints, and, more recently, in bioenergy applications as biofuels (Dyer *et al.*, 2008; Moser, 2012). According to the USDA, the global oilseed production is forecast at 604 Mt for 2020–2021, and is expected to reach a market value of US$255.3 billion by 2023 (https://apps.fas.usda.gov/psdonline/circulars/oilseeds.pdf). Soybean is the major seed oil commodity, with a production of 336.11 Mtin 2019 (OECD-FAO, 2019). Because of the importance of soybean as an agricultural crop, obtaining new lines with modified oil content and quality has been a major objective of soybean genetic and breeding programs. This is particularly directed at improving its characteristics, satisfying the needs of the food industry while respecting health recommendations for vegetable oil consumption (Clemente and Cahoon, 2008). With respect to the optimal fatty acid composition of plant seed oils, each sector of the food industry has its own requirements. High amounts of polyunsaturated fatty acids, such as linoleic or linolenic acids, is a requirement of healthy diets because of their benefits for cardiovascular health (Lee and Lip, 2003). On the other hand, other food producers, such as bakeries, require highly saturated fatty acids because of the need for oil stable to oxidation and high temperature (Clemente and Cahoon, 2008). However, these saturated fatty acids do not fulfill cardiovascular health requirements in the same way. While palmitic acid (C16:0) has a negative impact on the lipoprotein and cholesterol blood profile (Mensink and Khatan, 1990), stearic acid (C18:0) does not alter blood cholesterol levels (Kris-Etherton *et al.*, 2005). Therefore, seed oils with high stearic and/or oleic acid content would have a great impact in the bakery and cooking industries.

Modifying plant seed oil content is not an easy task. This is due to the high complexity of the seed oil and fatty acid biosynthetic pathways, where many enzymes work in a coordinated manner in different plant tissues and organelles (see Box 1). Very often problems associated with metabolic bottlenecks limit the extent of the impact of genetic modification on fatty acid composition (Cahoon *et al.*, 2007). Moreover, as constituents of membrane lipids, modifications of the fatty acid content might result in alterations of plant growth, yield, or development, as well as plant responses to biotic or abiotic stresses in which fatty acids are directly or indirectly involved (Nischiuchi and Iba, 1998; Kachroo *et al.*, 2001; Iba, 2002). Conventional breeding techniques have proven successful in obtaining soybean lines yielding more protein or oil on a per hectare basis (Clemente and Cahoon, 2008). The advances in molecular biology techniques opened the door to specifically targeted genes or metabolic pathways for tailoring of the seed oil fatty acid composition (Jaworsky and Cahoon, 2003; Damude and Kinney, 2008). More recently, CRISPR/Cas9 [clustered regularly interspaced palindromic repeats (CRISPR)/CRISPR-associated protein 9] technology has proven successful in modifying seed oil composition in several oil commodities such as rapeseed and soybean, increasing oleic acid content by editing the reticular omega-6 desaturase *FAD2* (Okuzaki *et al*, 2018; Do *et al.*, 2019). However, the difficulties of the application of CRISPR/Cas9 technology in plants with highly duplicated genomes, such as soybean, as well as the limitations for the commercialization of seeds from edited plants, particularly in the EU territory, mean that conventional genomic tools, such as TILLING, are still a powerful resource for soybean biotechnologists.

Box 1. **Complexity of the fatty acid biosynthesis pathway involves variability in metabolic interactions and differing organelle and tissue regulation**

Two different plant cell organelles, the plastid and the endoplasmic reticulum (ER), are involved in the biosynthesis of lipids and fatty acids. The initial steps take place in the plastid where the acetyl-CoA carboxylase (ACC) catalyzes the carboxylation of acetyl-CoA molecules to synthetize malonyl-CoA. This malonyl-CoA is used by the fatty acid synthase complex (FAS) in a series of condensation reactions that elongate the fatty acid chain. During these condensation reactions, the forming acyl chains are rapidly esterified to the acyl carrier protein (ACP). Finally, KASI condenses another malonyl-CoA molecule to obtain palmitoyl-ACP (16:0-ACP). After this, 16:0-ACP can undergo an additional condensation by KASII to obtain stearoyl-ACP (18:0-ACP). Still in the plastid, 18:0-ACP can be desaturated by the stearoyl-ACP desaturase (SACPD) to obtain oleyl-ACP (18:1-ACP). Five *GmSACPD* genes are present in the soybean genome, although only four of them seemed to be functional (Lakhssasssi *et al*., 2020); bold letters indicate the novel mutants in the *GmSACPD* family identified in this work. A portion of each 16:0-ACP, 18:0-ACP, and 18:1-ACP pool can be incorporated into galactolipids and phosphatidylglycerol in the plastid at the *sn*-1 and *sn*-2 positions of the glycerol backbone. Once incorporated, they can be substrates of omega-6 and omega-3 desaturases, *Gm*FAD6 and *Gm*FAD7/FAD8, to produce 18:2 and 18:3 fatty acids, respectively. In Arabidopsis, FAD7 and FAD8 enzymes produce both 16:3 and 18:3 omega-3 fatty acids and are encoded by single genes. On the contrary, in soybean, *Gm*FAD7/*Gm*FAD8 desaturases are multigenic families that only synthetize 18:3 in the plastid. A portion of each 16:0-ACP, 18:0-ACP, and 18:1-ACP pool is exported from the plastid to the ER in the form of acyl-CoA moieties and can be esterified to phospholipids (mostly phosphatidylcholine) where *Gm*FAD2 and *Gm*FAD3 desaturases synthesize 18:2 and 18:3 fatty acids, respectively. On the other hand, a portion of the acyl-CoA moieties can be incorporated into triacylglycerol (TAG) biosynthesis in the seed via the so-called Kennedy pathway. TAG is the major lipid fraction in plant seed oils. Several isoforms encode the *Gm*FAD2 and *Gm*FAD3 families in soybean. Two *Gm*FAD2-1 isoforms are seed specific, while *Gm*FAD2-2 is constitutively expressed in the whole plant. Three *Gm*FAD3 genes are encoded by the soybean genome with differential expression during seed development or cold temperature exposure (Román *et al.*, 2012). Fatty acid flux between the plastid and the ER, and vice versa, occurs and may vary among the different plant species. 18:3 Fatty acids synthesized in the plastid, mostly by *Gm*FAD7, act as precursors for the biosynthesis of jasmonates and, therefore, are likely to be involved in defense responses and biotic stress signaling. Genes in gray letters indicate low expression or inactive isoforms.

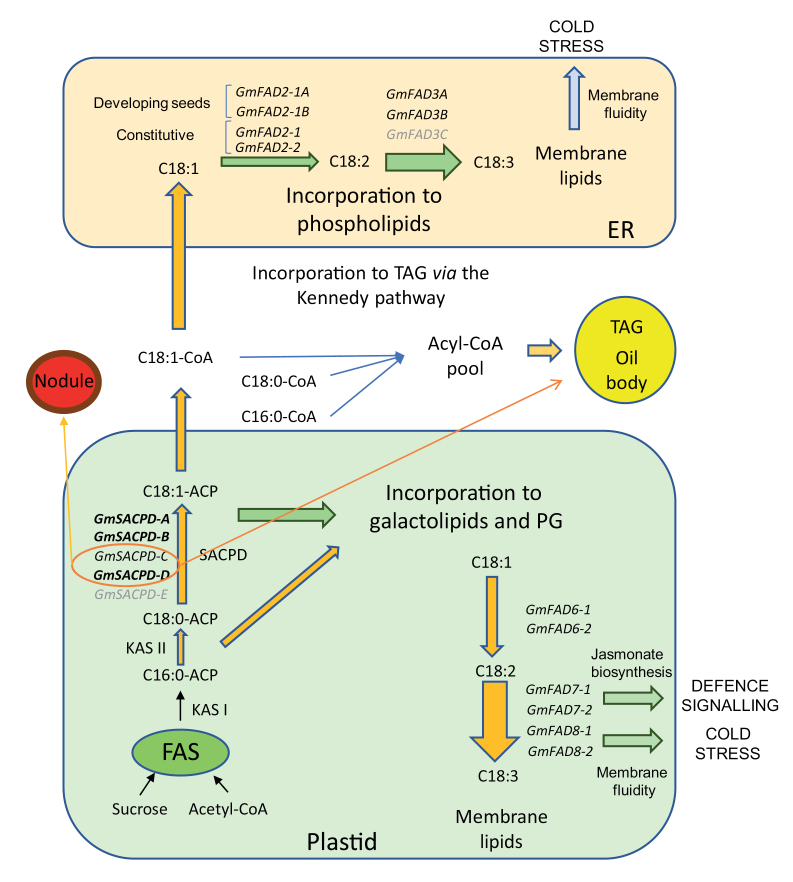

## TILLING-by-sequencing^+^ as an alternative to identify new soybean *GmSACPD* alleles that do not limit plant yield

In this work, Lakhssasi *et al*. (2020) aimed to identify new mutations at the *GmSACPD* gene family that might result in higher stearic acid content in the seed oil without affecting nodulation and plant growth. Previously characterized mutants in the *GmSACPD* gene showed poor germination and low seed yield (Zhang *et al.*, 2008; Gillman *et al.*, 2014). In previous work, Lakhssassi *et al.* (2017) described the existence of leaf structure and morphology changes as well as alterations in nodule structure in *GmSACPD-C* mutants. Despite the fact that five members of the *GmSACPD* gene family have been identified in the soybean genome, only mutations in the *GmSACPD-C* gene had been isolated in the previously characterized mutants (Zhang *et al.*, 2008; Gillman *et al.*, 2014; Lakhssassi *et al.*, 2017).

In this latest work, Lakhssassi and co-workers compared conventional gel-based TILLING with new TILLING-by-target capture sequencing. This is an extension of the TILLING-by-sequencing method that uses specific probe design to enrich the mutant population in a desired gene; in this case, the *GmSACPD* gene family. While gel-based TILLING identified no mutants, TILLING-by-target capture sequencing enabled the identification of 44 mutants, and 20 of them were further segregated. According to the authors, TILLING-by-target sequencing was 11 times more powerful, and 80% more successful, in the identification of true mutants when compared with other conventional TILLING techniques. This improvement in the number of mutants available for segregation resulted in the identification of novel mutations in the *GmSACPD-A*, *GmSACPD-B*, and *GmSACPD-D* genes, distinct from the previously characterized *GmSACPD-C* mutants that were previously identified using a forward genetics approach (Zhang *et al.*, 2008; Gillman *et al.*, 2014; Lakhssassi *et al.*, 2017).

Interestingly, while mutants in the *GmSACPD-C* genes showed deleterious effects on plant growth or nodule function, the novel mutants isolated in the *GmSACPD-A*, *GmSACPD-B*, and *GmSACPD-D* genes showed no effect on growth or nodulation. This is not striking given the relative transcript abundance of *GmSACPD-C* in the different soybean tissues. Lakhssassi *et al.* (2020) have shown that nodulation was not affected in *GmSACPD-A*, *GmSACPD-B*, and *GmSACPD-D* mutants due to their relatively low expression in the nodule, when compared with *GmSACPD-C* (fig. 7 in Lakhssasi *et al*., 2020). Whilst the expression of the *GmSACPD-C* gene was the highest in all plant tissues analyzed, including leaves, roots, seeds, and nodules, *GmSACPD-D* expression was the lowest (fig. 7 in Lakhssassi *et al.*, 2020). However, mutations in the *GmSACPD-A*, *GmSACPD-B*, and *GmSACPD-D* genes resulted in a 2- to 3-fold increase in stearic acid content. This result indicated that, despite its low expression levels, *GmSACPD-A*, *GmSACPD-B*, and *GmSACPD-D* contributed to 18:1-acyl carrier protein (ACP) levels in the soybean seed. Furthermore, this result suggested the existence of additional control points of its activity. In this sense, it is interesting to point out the lack of compensatory responses of the rest of the *GmSACPD* family members in the different mutant lines upon the mutation of a specific copy. Lakhssassi *et al.* (2020) have shown clearly the absence of any functional redundancy between the four *GmSACPD* gene family members in the synthesis of oleic acid from stearic acid. A possible explanation would be the presence of specific control mechanisms acting on each member of the *GmSACPD* family. This might suggest a high degree of specialization between gene family members, as has been already described for other desaturases in soybean (Román *et al.*, 2012). In fact, this high degree of specialization and specific control between the different members of multigenic families has been pointed out as a possible explanation of why highly duplicated genes were conserved during evolution in the soybean genome (Schmutz *et al.*, 2010).

In conclusion, the results from Lakhssassi and co-workers show how powerful the joint utilization of new sequencing technologies with more conventional ones such as TILLING can be. This is especially true in plant species with complex and highly duplicated genomes like soybean. Thus TILLING-by-target capture methodology greatly increases the possibility to identify more mutants, providing new materials for further characterization, and increasing the possibility of identifying novel phenotypes. This would be particularly useful for the identification of metabolic bottlenecks or differently genetically regulated genes (like the *GmSACPD* family), providing new information on the functioning of enzymes that could be used by breeders or biotechnologists to overcome undesired phenotypes that dramatically limit the applications of genetic technologies.
